# 41502 Does dietary fat composition predict short-term elevations in lipid levels in adults on a modified Atkins diet?

**DOI:** 10.1017/cts.2021.490

**Published:** 2021-03-30

**Authors:** Tanya J. W. McDonald, Bobbie J. Henry-Barron, Diane Vizthum, Mackenzie C. Cervenka

**Affiliations:** 1 Johns Hopkins University School of Medicine; 2 Johns Hopkins University Institute for Clinical and Translational Research

## Abstract

ABSTRACT IMPACT: Our work provides guidance on whether dietary fat intake influences serum cholesterol levels in response to ketogenic diet therapy in adults with epilepsy. OBJECTIVES/GOALS: The modified Atkins diet (MAD) is used in the management of drug-resistant epilepsy in adults. Some patients on MAD show an increase in serum levels of total cholesterol and low-density lipoprotein (LDL) cholesterol. We explored whether dietary fat composition predicts short-term elevations in serum lipid levels in diet-naive adults who begin MAD. METHODS/STUDY POPULATION: Participants self-reported their diet intake with 3-day food records at baseline, 1 month and 2 months. Food records were analyzed using Nutrition Data System for Research software. Fasting serum levels of total cholesterol (TC), high-density lipoprotein (HDL) cholesterol, and triglycerides were also collected and LDL level calculated at baseline, 1 month, and 2 months. RESULTS/ANTICIPATED RESULTS: 38 patients submitted complete food records at each study visit (baseline, 1 month, and 2 month). Compared to baseline diet intake, there was a significant reduction in daily carbohydrate intake at 1 and 2 months (p<0.001) and a significant increase in daily fat intake at 1 and 2 months (p<0.001). There was also a significant increase in daily saturated fatty acid (SFA) intake at 1 and 2 months (p<0.001), daily mono-unsaturated fatty acid (MUFA) intake at 1 and 2 months (p<0.001), and daily cholesterol intake at 1 month (p<0.05) and 2 months (p<0.001), but no change in daily poly-unsaturated fatty acid (PUFA) intake over time. Compared to baseline, there was a significant increase in serum LDL at 1 month (p<0.001) and 2 months (p<0.01) and an increase in serum TC at 1 month (p<0.01) but not 2 months. DISCUSSION/SIGNIFICANCE OF FINDINGS: Despite a significant increase in total fat, saturated fat and mono-unsaturated fat intake as well as an increase in total cholesterol and LDL levels following MAD initiation, dietary fat composition appears to minimally predict serum lipid values in the short term.

